# Centralization Within Sub-Experiments Enhances the Biological Relevance of Gene Co-expression Networks: A Plant Mitochondrial Case Study

**DOI:** 10.3389/fpls.2020.00524

**Published:** 2020-06-04

**Authors:** Simon R. Law, Therese G. Kellgren, Rafael Björk, Patrik Ryden, Olivier Keech

**Affiliations:** ^1^Department of Plant Physiology, Umeå Plant Science Centre, Umeå Universitet, Umeå, Sweden; ^2^Department of Mathematics and Mathematical Statistics, Umeå Universitet, Umeå, Sweden

**Keywords:** correlation, gene co-expression network, metabolism, method, plant mitochondria

## Abstract

Gene co-expression networks (GCNs) can be prepared using a variety of mathematical approaches based on data sampled across diverse developmental processes, tissue types, pathologies, mutant backgrounds, and stress conditions. These networks are used to identify genes with similar expression dynamics but are prone to introducing false-positive and false-negative relationships, especially in the instance of large and heterogenous datasets. With the aim of optimizing the relevance of edges in GCNs and enhancing global biological insight, we propose a novel approach that involves a data-centering step performed simultaneously per gene and per sub-experiment, called centralization within sub-experiments (CSE). Using a gene set encoding the plant mitochondrial proteome as a case study, our results show that all CSE-based GCNs assessed had significantly more edges within the majority of the considered functional sub-networks, such as the mitochondrial electron transport chain and its complexes, than GCNs not using CSE; thus demonstrating that CSE-based GCNs are efficient at predicting canonical functions and associated pathways, here referred to as the core gene network. Furthermore, we show that correlation analyses using CSE-processed data can be used to fine-tune prediction of the function of uncharacterized genes; while its use in combination with analyses based on non-CSE data can augment conventional stress analyses with the innate connections underpinning the dynamic system being examined. Therefore, CSE is an effective alternative method to conventional batch correction approaches, particularly when dealing with large and heterogenous datasets. The method is easy to implement into a pre-existing GCN analysis pipeline and can provide enhanced biological relevance to conventional GCNs by allowing users to delineate a core gene network.

**Author Summary:**

Gene co-expression networks (GCNs) are the product of a variety of mathematical approaches that identify causal relationships in gene expression dynamics but are prone to the misdiagnoses of false-positives and false-negatives, especially in the instance of large and heterogenous datasets. In light of the burgeoning output of next-generation sequencing projects performed on a variety of species, and developmental or clinical conditions; the statistical power and complexity of these networks will undoubtedly increase, while their biological relevance will be fiercely challenged. Here, we propose a novel approach to generate a “core” GCN with enhanced biological relevance. Our method involves a data-centering step that effectively removes all primary treatment/tissue effects, which is simple to employ and can be easily implemented into pre-existing GCN analysis pipelines. The gain in biological relevance resulting from the adoption of this approach was assessed using a plant mitochondrial case study.

## Introduction

Over the last two decades, the growth of available transcriptome data in an increasing number of species has given rise to a multitude of gene co-expression networks (GCNs). By constructing these networks on data sampled from diverse developmental processes, tissue types, pathologies, mutant backgrounds, or stress conditions; researchers can better comprehend the physiological and molecular pathways that underpin complex biological systems (Carrera et al., 2009; Emmert-Streib et al., 2014; Liesecke et al., 2018; Castro et al., 2019). These networks rely on mathematical approaches to identify causal relationships in gene expression dynamics and the most prevalent are those based on undirected correlation approaches, such as Pearson correlation coefficient, Spearman’s rank correlation coefficient, partial correlation, or biweight midcorrelation (Langfelder and Horvath, 2008; Song, 2012).

For experiments where the number of genes greatly exceeds the number of samples, it is common to assume that the network is *sparse*, i.e. the most pronounced correlations are concentrated within sub-networks. A number of shrinkage techniques for estimating correlations in sparse networks have been proposed (Friedman et al., 2000, 2008, 2014; Schäfer and Strimmer, 2005; Wang and Huang, 2014). Independent of the approach, the resulting correlation matrix is commonly used to construct an adjacency matrix (a 0 to 1 matrix where edges are indicated by the presence of a “1”), which defines an *unweighted* network. These conventional correlation methods have been demonstrably successful at identifying cohorts of strongly co-expressed genes, and thus have been used extensively in the generation of GCNs. However, these methods also have their disadvantages. This is especially apparent with large and heterogenous datasets, in which a substantial fraction of the predicted correlations are expected to be statistically significant, and causal gene-to-gene connections are obscured by the overwhelming presence of false-positives and false-negatives. Non-causal relationships can arise from indirect connections with other gene products (i.e. an edge between two genes via a gene-intermediate) and from non-biological sources such as influences resulting from experimental design. Therefore, validation of GCNs can be challenging as there are only a limited number of gene-to-gene relationships (positive or negative) experimentally demonstrated (Qian and Dougherty, 2013; Chai et al., 2014; Banf and Rhee, 2017). Partial correlation is a standard approach used to attenuate non-causal relationships generated by the influence of other genes. One such approach, Gaussian Graphical Modeling (GGM), is commonly used to interrogate the direct association between two genes, independent of the effects of surrounding genes present in the dataset. A number of thorough GGM studies in the model plant species *Arabidopsis thaliana* (Arabidopsis) have demonstrated the statistical power of this technique, both for selected pathways and on a genome-wide scale (Wille et al., 2004; Ma et al., 2007, 2015). Yet, since the biological relevance of an edge linking two nodes in such networks can be called into question, a complementary approach is to base the validation (i.e. the biological relevance of the output) on physical and functional proximity, arguing that the fraction of causal relationships should be relatively high within sets of genes encoding proteins that are part of the same complex or are involved in the same metabolic pathway.

GCNs are commonly constructed in four steps, which include: (i) data pre-processing, (ii) estimation of pair-wise associations, (iii) prediction of the network, and (iv) identification of the sub-networks in the network (van Dam et al., 2018). For the pre-processing step, several approaches to alleviate the potential heterogeneity between the samples have been proposed. For instance, batch-effect removal approaches effectively eliminate the systematic, technical errors inherent to multi-experiment comparisons (Chen et al., 2011; Nygaard et al., 2016). An alternative approach is to split the heterogenous data into more homogenous subsets (e.g. into tissue/treatment/stress specific datasets) and to construct set-specific networks that are later merged into a consensus network (Langfelder and Horvath, 2008; Wren, 2009). However, in splitting the data a trade-off can arise between subset sample size and the resulting subset homogeneity. Despite these alternatives, GCNs obtained utilizing partial correlation, batch-effect removal approaches, or subset division will not reduce non-causal relationships resulting from unquantifiable factors, e.g. treatment/tissue effects between samples. Hence, there is currently a lack of methodology to robustly derive informative GCNs from complex datasets generated by heterogeneous experiments.

With the aim of optimizing the biological relevance of edges in GCNs and enhancing global biological insight, we challenged different methodologies in the generation of these networks by using, as a case study, a subset of nuclear genes encoding proteins targeted to the plant mitochondrion (as defined in Chrobok et al., 2016). To achieve this, we applied a novel pre-processing step that we call centralization within sub-experiments (CSE), which reduces the impact of the confounding effects of treatment-induced and tissue-specific responses. In contrast to conventional batch-effect removal approaches, the CSE step is applied to datasets at the level of biological replicates derived under the same experimental conditions. Hence, CSE also removes technical bias introduced by variability between experiments. Here, we compared several widespread GCN approaches with, or without the CSE pre-processing step. Biological validation was conducted by categorizing a subset of genes encoding for plant mitochondrial proteins with respect to expression patterns, functional proximity, and functional categories. CSE combined with GCN (utilizing Pearson correlation) provided the optimum balance for the ease of data processing vs. the utility of the output. Consequently, a mitochondrial network based on CSE Pearson correlation was selected for further downstream applications of the method.

## Results

To gain clarity, this results section has been divided in three parts: Methodology, Validation, and Application.

### Methodology

#### Definition of the Problem

**FIGURE 1:**
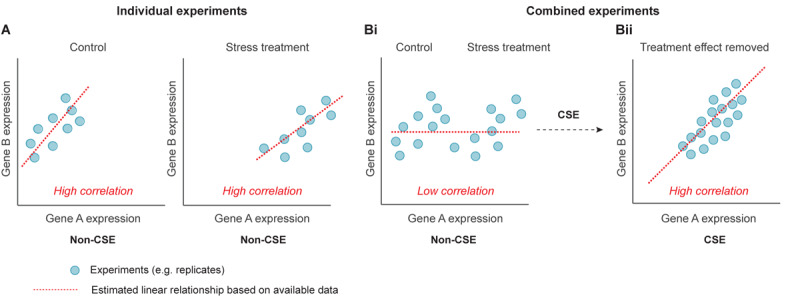
Schematic illustrating the utility of centralization within sub-experiments (CSE) when comparing genes from a diverse background of treatments. **(A)** Conventional correlation analysis of two genes (Gene A and Gene B) under control conditions reveals a high positive correlation. Corresponding correlation analysis of the same two genes in response to a stress treatment again reveals a high positive correlation. **(Bi)** When both the control and stress experiments are combined, conventional correlation analysis results in a low level of correlation (false negative). **(Bii)** By carrying out CSE, the mean effect between replicates is removed, and subsequent conventional correlation analysis now reveals the “core” high correlation between Gene A and Gene B.

We consider a problem where we have gene expression data from a large number of diverse experiments, e.g. experiments from different tissues, treatments, and developmental stages. The objective is to predict the edges of an undirected graph with *n* nodes (i.e. genes), where an edge represents the most pronounced co-expression between a pair of genes. Often, the level of co-expression between genes will be context-dependent, e.g. tissue, growth condition or developmental stage (Figure 1). Here, we are primarily interested in detecting the core network, i.e. to estimate the co-expression between genes that are prominent in the majority of the considered sub-experiments. A sub-experiment is defined as a set of assays derived under “identical settings”, i.e. the assays within the sub-experiment can be treated as biological replicates. We thus propose a pre-processing step (CSE) that enables prediction of the core network.

#### Centralization Within Sub-Experiments

We consider normalized gene expression data from *s* sub-experiments, i.e.

$$\left\{x_{i\,j\,k}\right\},\,i=1,\ldots ,n,\,j=1,\ldots ,s,\,k=1,\ldots ,r_{j},$$

where *x*_*ijk*_ denotes the normalized gene expression for gene *i* observed on the *k*^*th*^ biological replicate in sub-experiment *j*. CSE is a simple pre-processing step whereby mean-centralization within sub-experiments is applied to each gene separately, i.e. the CSE-processed expressions are obtained as:

$$x_{i\,j\,k}^{C\,S\,E}=x_{i\,j\,k}-{\bar{x}}_{ij.},$$

where ${\bar{x}}_{ij.}$ denotes the mean-expression of gene *i* in the *j*^*th*^ sub-experiment, *i* = 1, …, *n, j* = 1, …, *s, k* = 1, …, *r_*j*_.*

It should be noted that the mean value of the centralized data within a sub-experiment will always be zero. Thus, CSE negates pronounced correlations driven by differences between the sub-experiments. For example, a given stress may induce gene expression in genes that are expressed in “independent” pathways resulting in false-positive and false-negative predictions (Figure 1 and Supplementary Figure S1).

#### Construction of Gene Co-expression Networks

Gene co-expression networks can be constructed in various way, but we selected commonly used approaches to assess the effect of CSE application. The GCNs were constructed in a three-step procedure: (i) the pre-processed dataset was either centralized using CSE (CSE) or not centralized (non-CSE), (ii) pairwise correlations were calculated using either Pearson correlation or partial correlation, and (iii) the *sign matrix* (i.e. an adjacency matrix whose entries are either 1 or 0) was constructed by controlling the fraction ω of edges at a desired level, i.e. controlling the sparsity at level ω. The network was defined by the output of the adjacency matrix; where a “1” represents an edge corresponding to a level of the absolute co-expression value between genes that satisfies a given cut-off. In this study, four different principal networks were evaluated: combining CSE and Pearson correlation (CSE Pearson correlation), CSE and partial correlation (CSE partial correlation), and Pearson and partial correlation applied in the absence of CSE (non-CSE Pearson correlation and non-CSE partial correlation, respectively). In addition to the four main networks described above, a further comparison was performed using two permutations of a commonly used networking approach, known as weighted gene correlation network analysis (WGCNA) (Zhang and Horvath, 2005). To that end, we constructed: (i) a network based on all data (WGCNA All) and (ii) a consensus network based on four tissue-specific sub-networks (WGCNA Consensus). In both cases, networks were prepared using either non-CSE or CSE data (cf. “Materials and Methods” section). Furthermore, a final comparison was conducted introducing two additional methods, BC3Net (de Matos Simoes and Emmert-Streib, 2012) and GeneNet (Schäfer et al., 2001) with CSE and non-CSE data. The sparsity of all GCNs was controlled at ω = 0.005 and the Walktrap community detection algorithm (Pons and Latapy, 2005) was used to identify communities in the predicted GCN based on Pearson correlation. The objective here was not to predict all edges in the core network, but to predict the most pronounced edges, which justifies the use of an arbitrary chosen threshold. Moreover, having the same sparsity in all predicted networks simplified the validation steps as described below.

Applying the conceptual reasoning outlined above on a network using simulated data demonstrated that CSE partial correlation removes non-causal edges arising from the influence of other genes and non-causal edges caused by external factors (Supplementary Figure S1). Similar results were obtained for CSE Pearson correlation, with the exception that a few false, but relatively weak, edges appeared. The network utilizing non-CSE data in tandem with Pearson correlation, arguably the most standard approach, resulted in dense networks with multiple false positives. Due to computational constraints, partial correlation approaches may not be suitable for constructing GCNs when the number of genes is much larger than the number of experiments (see the section “Discussion”).

#### Evaluation of Gene Co-expression Networks

We consider a core network *C*, with *n* nodes and *k* edges, where the edges correspond to the fraction ω of the strongest co-expression correlation. A sub-network *A ⊂ C*, with *n*_*A*_ nodes and *k*_*A*_ edges is said to be pronounced if *k*_*A*_ is larger than the expected number of edges in a randomly selected sub-network with *n*_*A*_ nodes, i.e.

$$k_{A}>\omega \,\left(\begin{array}{c} n_{A}\\ 2 \end{array}\right).$$

The network *C* is commonly unknown, but it may still be possible to identify several pronounced sub-networks, e.g. by considering physical or functional proximity [see the section “Preparing Elements of the Mitochondrial Working Model” (iii, iv)].

We propose that the relative performance of predicted GCNs, all with the same sparsity ω, can be evaluated based on the observed number of edges within defined sub-networks. In short, we argue that the more observed edges (the lower *P*-values) within sub-networks, the better the predicted networks are (see “Materials and Methods” for further details). With that being said, there is a risk to overestimate the number of edges within the sub-networks resulting in an incorrect ranking of the considered networks; however, this risk decreases as the number of sub-networks is increased.

### Validation

For this study, we chose the plant mitochondrion as a focal point for three reasons: (i) assessing the biological relevance of our findings became much easier due to our pre-existing knowledge of plant mitochondrial metabolism, (ii) the number of genes to work with is low (ca. 1000 nuclear genes coding for mitochondrial-targeted proteins), hence easing the application of partial correlation methods, and (iii) the interest in mitochondrial biology is strong, as this organelle is recognized as a central energetic, signaling, and stress response hub in most eukaryotic cells.

#### The Effect of Tissue Type on Gene Co-expression Networks

**FIGURE 2:**
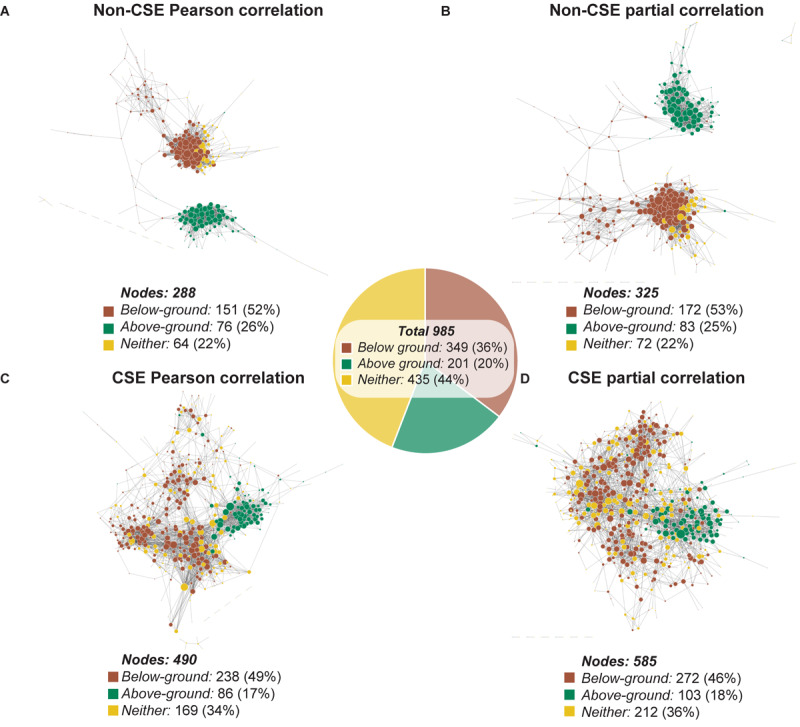
Visualization of the mitochondrial network using four different pre-processing and correlation approaches. A manually curated mitochondrial gene list was cross-referenced with the AtGenExpress Expression Atlas spanning different tissues, developmental stages, and stresses (Schmid et al., 2005; Kilian et al., 2007; Goda et al., 2008). This data was either subject to CSE or left unprocessed, prior to correlation analysis using either Pearson correlation or partial correlation. Each of the four resulting networks was visualized using Cytoscape. For each network, only nodes with at least one edge to another node were included. Each node (gene) was colored based on their classification as either below-ground dominant (brown), above-ground dominant (green) or dominance in neither tissue (yellow). The diameter of each node is proportional to the number of edges it has to a neighboring node. **(A)** Network of non-CSE Pearson correlation. **(B)** Network of non-CSE partial correlation. **(C)** Network of CSE Pearson correlation. **(D)** Network of CSE partial correlation.

Visualization of the four GCNs generated using Cytoscape (organic layout; Shannon et al., 2003) revealed networks that shared strong similarities in structure depending on whether CSE was applied or not (Figure 2 and Supplementary Table S2). Those networks based on non-CSE data displayed two distinct primary clusters of nodes (Figures 2A,B), while those based on CSE data were more integrated (Figures 2C,D). To uncover the source of these distinct clusters in the non-CSE data, we returned to the original data from the AtGenExpress expression atlas, and defined each gene as presenting dominant expression in either below-ground tissues (e.g. roots) or above-ground photosynthetic tissues (e.g. shoots and leaves) (see the section “Materials and Methods” for details). Using these definitions, nodes (genes) from the networks were colored based on their classification as either below-ground dominant (brown), above-ground dominant (green) or dominance in neither tissue (yellow) (Figure 2). This rapidly demonstrated the strong influence tissue-of-origin has over the resulting GCN, and the efficacy of CSE in resolving this. Notably, in addition to the increased integration of genes with different tissue-dominances, the number of nodes with edges to other nodes was significantly (*P* < 0.0001; Fisher’s exact test) larger following CSE. Here, the null hypothesis was that the number of nodes with edges was the same for networks derived using CSE or not using CSE. Furthermore, the distribution of genes with tissue-dominance established an increased inclusion of genes with no tissue dominance (Neither), which brought these networks closer to the native distribution of tissue of origin dominance observed in the total set. This suggests that by removing external biases, CSE of data could introduce a wider cross-section of genes into a GCN and thus reveals novel interactions.

#### Assessing Interactions Based on Functional Proximity

**FIGURE 3:**
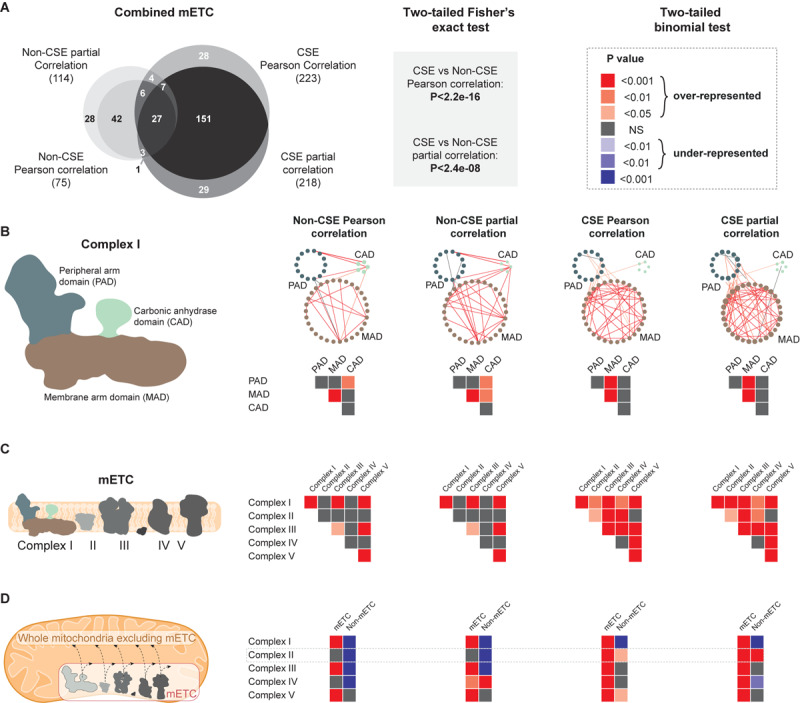
Comparative analysis of four different correlation methods in defining interactions based on functional proximity. The following gene subsets of the mitochondrial electron transport chain were analyzed using non-CSE Pearson correlation, non-CSE partial correlation, CSE Pearson correlation and CSE partial correlation. *P*-values were calculated (two-tailed binomial test) for the probability associated with the expected vs. observed number of edges and a color-grading scheme of the resulting *P*-values applied. **(A)** A Venn diagram illustrating the overlap of connections between the complexes of the mitochondrial electron transport chain (mETC), when analyzed using the four different correlation methods. A two-tailed Fisher’s exact test was employed to test the significance of the difference in the number of edges (derived from connections within and between the five complexes of the mETC) in the networks based on non-CSE vs. CSE data. **(B)** The significance of the edges between the three domains of Complex I. **(C)** The significance of the edges within a given complex or between the different complexes of the ETC. **(D)** Between the individual complexes of the mETC vs. the unified mETC or the rest of the mitochondrial set excluding the mETC.

Our first approach at challenging the four main different GCNs was to examine the resulting distribution of edges upon a small isolated subset of the mitochondrial network, encoding components of the mitochondrial electron transport chain (mETC). The mETC is central to the bioenergetic function of mitochondria and the array of genes that comprise its five complexes have been demonstrated to be expressed at relatively stable levels in a variety of tissue types and developmental stages (Lee et al., 2011). In a first step, a comparison between the mETC set isolated from four networks (non-CSE/CSE, Pearson or partial correlations, respectively) revealed a significantly (*P* < 0.0001; two-tailed Fisher’s exact test) higher number of edges (derived from connections within and between the five complexes of the mETC) in the networks based on CSE data, while the influence of partial correlation vs. Pearson correlation was comparatively small (Figure 3A). Similarly, two WGCNA approaches (i.e. All and Consensus; cf. “Materials and Methods”) demonstrated that the resulting networks based on CSE data detected significantly (*P* < 0.0001) more edges within and between the five complexes of the mETC than in non-CSE networks (Supplementary Figure S2A). Of note, the non-CSE networks detected very few edges, with zero edges detected for non-CSE WGCNA All and only one edge for non-CSE WGCNA Consensus.

As the same sparsity is applied to all approaches, the total number of edges in the entire network is held consistent between them. Thus, the enrichment of edges within the mETC observed here represents a valuable indication of putative biological interaction. Our next step was to assess the distribution of edges within a single complex of the mETC. The NADH dehydrogenase, commonly known as Complex I, is composed of three domains: the peripheral arm domain (PAD), the membrane arm domain (MAD), and the carbonic anhydrase domain (CAD) (Peters et al., 2013). In turn, each domain is composed of an assembly of proteins that carry out highly specialized functions, and thus proved ideal to assess the relevance of the distribution of edges between the different approaches. Similar to the distribution of edges for the entire mETC, networks prepared using CSE data showed a greater number of edges within PAD and MAD domains as well as between them (Figure 3B, Supplementary Figure S2B, and Supplementary Table S3). Yet, the number of edges between CAD and PAD/MAD domains became non-significant when data were CSE pre-processed (Figure 3B, Supplementary Figure S2B, and Supplementary Table S3). As a matter of fact, CAD appears to have a function independent of the primary role of Complex I, which is the oxidation of NADH and the transfer of electrons to the pool of ubiquinone. Indeed, a recent study has reported that CAD may have a supporting role in Complex I assembly, rather than a direct enzymatic function (Fromm et al., 2016). When this examination was expanded to look at the distribution of edges within and between all five complexes of the mETC, a similar enrichment of significant interactions was observed with the CSE data, but not with the non-CSE data (Figure 3C and Supplementary Figure S2C). Interestingly, when the distribution of edges between individual complexes and either (i) pooled complexes of the mETC, or (ii) the rest of mitochondrial set (total mitochondrial set, excluding the mETC), the networks based on non-CSE data showed relatively poor correlations with the pooled mETC and even weaker connections with the non-mETC components (Figure 3D and Supplementary Figure S2D). In contrast, the CSE data showed significant (*P* < 0.001; two-tailed binomial test) connections between the individual complexes and the pooled mETC, with weaker connections to the non-mETC components. One important exception to this was the significant (*P* < 0.01 in CSE Pearson correlation, and *P* < 0.001 in CSE partial correlation) connection observed between Complex II and the non-mETC components. Notably, Complex II (also called succinate dehydrogenase) lies at the confluence of two essential bioenergetic functions of the mitochondrion: the mETC and the TCA cycle. As such, it is particularly notable that the CSE data (although not for WGCNA networks) identified Complex II as having significant interaction with non-mETC components. Examination of the composition of edges between Complex II and these non-mETC genes revealed that they were indeed significantly (*P* < 0.0001) enriched in components of the TCA cycle. Taken together, these observations strongly support that CSE of data prior to correlation analysis can reveal gene-to-gene interactions indicative of highly valuable biological relationships such as association to shared protein domains or consecutive enzymes in a metabolic pathway. Furthermore, Pearson or partial correlations seem to provide a better biological insight than the two weighed networks.

#### Assessing Interactions Based on Connectivity Within and Between Mitochondrial Functional Categories

**FIGURE 4:**
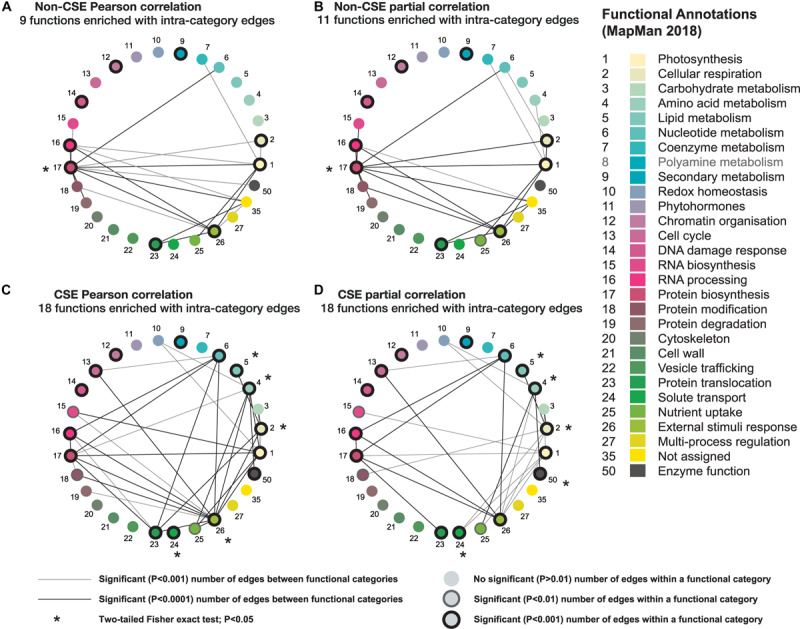
Comparative analysis of four different correlation methods based on connectivity between different functional categories in mitochondria. Using newly updated MapMan annotations (MapMan X4 Release 1.0, 2018; Usadel et al., 2009), the mitochondrial set was subdivided into 29 different functional categories. Only functional categories with at least one significant (*P* < 0.0001; one-tailed binomial test) connection to another category are displayed for each method. Nodes with a black outline indicate functional categories with significant (*P* < 0.001) intra-connectivity, nodes with a faint outline indicates functional categories with significant (*P* < 0.01) intra-connectivity, nodes lacking an outline indicates functional categories that do not have a significant (*P* > 0.01) number of edges within a functional category. Lines between functional categories indicate a significant (*P* < 0.0001) number of edges exist between the genes comprising both function categories. The presence of an * indicates that there is a significantly (*P* < 0.05; two-tailed Fisher’s exact test) greater proportion of edges when comparing CSE-processed data vs. non-CSE data. **(A)** Non-CSE Pearson correlation, **(B)** non-CSE partial correlation, **(C)** CSE Pearson correlation, and **(D)** CSE partial correlation.

Using the newly updated functional annotations established for the MapMan platform (MapMan X4 Release 1.0, 2018; Usadel et al., 2009), each gene of the mitochondrial set was assigned to one of 29 functional categories. By grouping genes belonging to the same functional categories, we were able to measure the number of edges between genes *within* a functional category, versus those *between* different, yet interrelated, functional categories (Figure 4). In brief, when CSE had been carried out (Figures 4C,D), the number of predicted edges between genes within the same category is much higher (nearly double; *P* < 0.01; one-tailed binomial test) than is observed when the data is non-CSE; also, direct intra-category comparison revealed significantly (*P* < 0.05; Fisher’s exact test) more edges within the majority of these CSE networks (Figures 4A,B). Additionally, in the two CSE datasets, the number of significant edges between different functional categories also increases, when compared to their non-CSE counterparts. These inter-category edges were often highly biologically relevant: for example, a significant (*P* < 0.0001) edge was observed between *nucleotide metabolism* and *protein biosynthesis* in each of the four methodologies (Figures 4A–D), which is hardly surprising given their canonic interconnectivity. In contrast, some connections were only observed in the case of the CSE datasets (Figures 4A,B), such as the significant (*P* < 0.0001) edges between *cellular respiration* and *carbohydrate* and *lipid metabolism*, as well as the connection between *protein biosynthesis and protein translocation*. For these processes to operate efficiently, a high level of coordination is required in the regulation of the genes involved, which supports these additional inter-category edges.

Furthermore, corresponding analyses of networks prepared using WGCNA All/WGCNA Consensus with or without CSE pre-processing revealed similar findings, though the enhancement provided by CSE appeared diminished in the case of WGCNA Consensus (Supplementary Figure S3). In summary, the known biological pathways strongly corroborate the input from the CSE co-expression data generated with our mitochondrial dataset and undoubtedly strengthen its consideration for future analyses. Following these validation steps, the negligible difference in results between CSE Pearson correlation, partial correlation, and WGCNA, contrasted with both time and computational demands, especially in the case of partial correlation. We therefore used only CSE Pearson correlation for the subsequent applications.

### Application

#### Using the Network to Predict the Function of Uncharacterized Mitochondrial Genes

**FIGURE 5:**
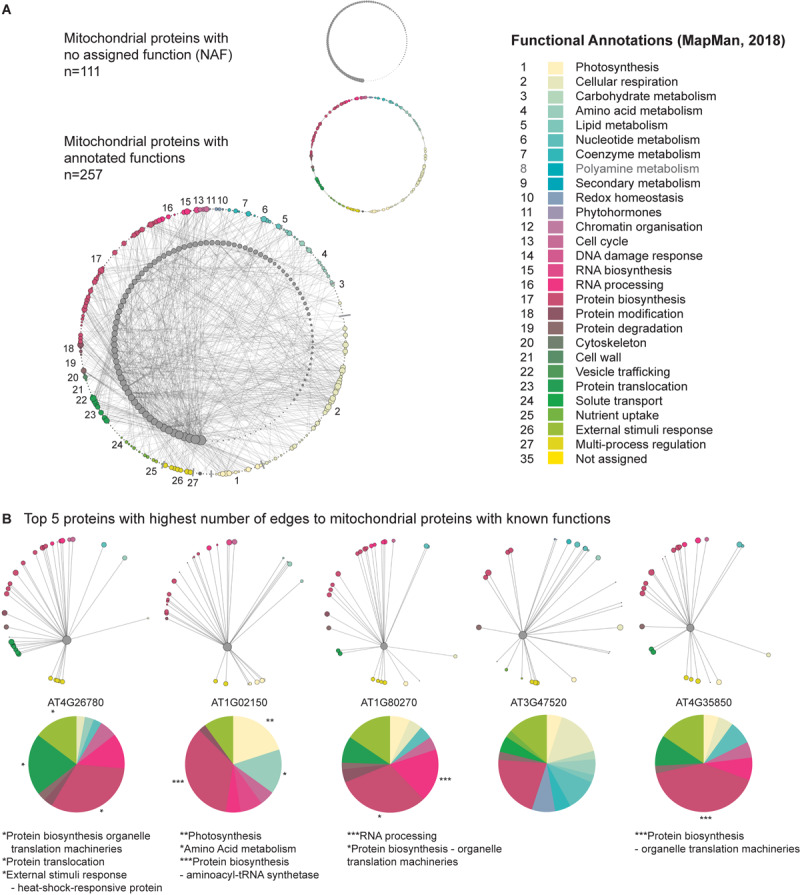
Identification of candidate functions for mitochondrial proteins with unknown functions. Pearson correlation was carried out on CSE pre-processed data spanning the mitochondrial set, over 370 unique conditions comprising the AtGenExpress Expression Atlas. Out of this list, a sub-population of genes was established which had unknown functional annotations. This sub-population was then analyzed to identify significant interactions with mitochondrial proteins with known functions, resulting in a suite of 109 mitochondrial proteins with unknown functions. By annotating the functional categories of the known mitochondrial genes, putative functional relationships can be assigned to these as yet uncharacterized proteins. **(A)** Network representation of the interactions between 109 mitochondrial proteins with no annotated functions and 248 mitochondrial proteins with known functions. **(B)** The five proteins with no annotated functions displaying the highest number of edges to the mitochondrial set are shown, with a functional breakdown of the distribution of edges. Significant over-representation of a given functional category was assessed using a *z*-score approach and the results have been marked with the following: *p < 0.05; **p < 0.01; ***p < 0.001.

The functional annotations applied to the genes comprising the mitochondrial network (introduced above) encompassed a subset of mitochondrial genes that at the time of the publication of the MapMan hierarchical set of functional categories (BINs; MapMan X4 Release 1.0, 2018), encoded proteins with *no assigned functions* (NAFs; Functional Category 35). This provided an ideal target group that we could systematically interrogate, in a “guilt by association manner,” to determine if their relationship to other genes of known functions could support their putative function. A subsequent mitochondrial network was established, which comprised 111 NAF genes and 257 mitochondrial genes encoding proteins with known functions that had at least one edge to a NAF gene (Figure 5A and Supplementary Table S4). The NAF genes were then arranged in descending order based on those with the greatest number of edges to genes with known functions. We then selected the top five NAF genes and identified the genes they interacted with. The distribution of their associated functional annotations was then assessed to discern if they were enriched in a particular function (Figure 5B).

The top five NAF genes displayed significant (following a *z*-score analysis) over-representations with a range of different functional categories. The NAF with the greatest number of connections with genes of known function, AT4G26780, had a significant enrichment of edges with (i) protein biosynthesis – organelle translation machineries (*P* < 0.05), (ii) protein translocation – TOM translocation and TIM insertion systems (*P* < 0.05), and (iii) external stimuli response – heat-shock-responsive protein (*P* < 0.05). Interestingly, this protein has been proposed to encode Mge2, which is one of two mitochondrial GrpE proteins in Arabidopsis. The remaining homolog, Mge1 serves as a co-chaperone alongside Hsp70, which together form a vital part of the presequence-assisted motor (PAM) complex that aids in the transport of precursor proteins through the TIM17:23 translocase (Hu et al., 2012; Ghifari et al., 2018). While Mge1 appears to have more constitutive house-keeping duties, Hu et al. (2012) demonstrated that Mge2 was specifically induced by heat and suggested that it could be required for mitochondrial protein import and folding during periods of heat stress, a hypothesis that appears to be supported by our GCN predictions. The second gene interrogated (AT1G02150), had a significant enrichment of edges with (i) photosynthesis functions (*P* < 0.01), (ii) amino acid metabolism (*P* < 0.05), and (iii) protein biosynthesis – aminoacyl-tRNA synthetase (*P* < 0.001). At present, little is known about this protein, however, the Arabidopsis Information Portal (Araport) 11 classifies it as belonging to the tetratricopeptide repeat (TPR)-like superfamily (Cheng et al., 2017). TPR domains can be found in a diverse number of proteins, where they mediate protein–protein interactions; particularly in the formation of protein complexes. The strong significant (*P* < 0.001) over-representation with aminoacyl-tRNA synthetase functions (and the weaker, though still significant over-representation of amino acid metabolism functions) observed here is particularly interesting, as there is evidence that TPR-containing proteins can act as interacting mediators and co-chaperones in the formation of aminoacyl-tRNA synthetases (Han et al., 2007; Kim et al., 2014); suggesting that this protein may have a role in assisting amino acid loading of tRNAs in Arabidopsis. The third gene interrogated (AT1G80270) had a significant enrichment of edges with (i) RNA processing (*P* < 0.001) and (ii) protein biosynthesis – organelle translation machineries (*P* < 0.05). Assessing the available literature, this protein has been reported as belonging to the pentatricopeptide (PPR) superfamily (Doniwa et al., 2010), which are predominately mitochondrial or plastid targeted proteins and have been demonstrated to have a diverse array of roles associated with RNA metabolism, such as RNA editing, splicing, stability, and translation (Barkan and Small, 2014). AT1G80270, known as PPR596, has been demonstrated to be involved in the C-to-U editing efficiency of ribosomal protein S3 (RPS3; AtMg00090), which is noteworthy as in our study, PPR596 was also significantly enriched in connections with organelle translation machinery functions (Doniwa et al., 2010). Regarding AT3G47520, despite the surprising lack of a proper annotation by Mapman, this gene had been characterized and encodes an isoform of the mitochondrial dehydrogenase (mMDH2; Tomaz et al., 2010; Lindén et al., 2016). Although no functional categories were enriched, the big proportion taken by the categories redox homeostasis, cellular respiration and protein biosynthesis strongly supports the physiological role of mMDH2. Finally, the protein encoded by AT4G35850 had a significant (*p* < 0.001) enrichment of edges with protein biosynthesis – organelle translation machineries (large and small mitoribosome subunit) functions. Very little is known about this protein, but it has been classified as belonging to the PPR superfamily by Araport11, and could thus have a similar role to that of PPR596; as an editing factor associated with the correct processing of transcripts encoding mitoribosomal subunits, or be associated with ribosomes in other ways described in the literature; such as maintaining the stability of assembled mito-ribosomes following translation (Schmitz-Linneweber and Small, 2008); or promoting translational initiation by selectively recruiting mitoribosomes to the start codon of their target transcripts (Manavski et al., 2012; Haïli et al., 2016). Taken together, these findings suggest that CSE pre-processing aids guilt-by-association analyses and offers an easy to implement first step in the process of characterizing genes with unknown functions.

#### Synergy of CSE Approaches in the Analysis of Plant Stress

**FIGURE 6:**
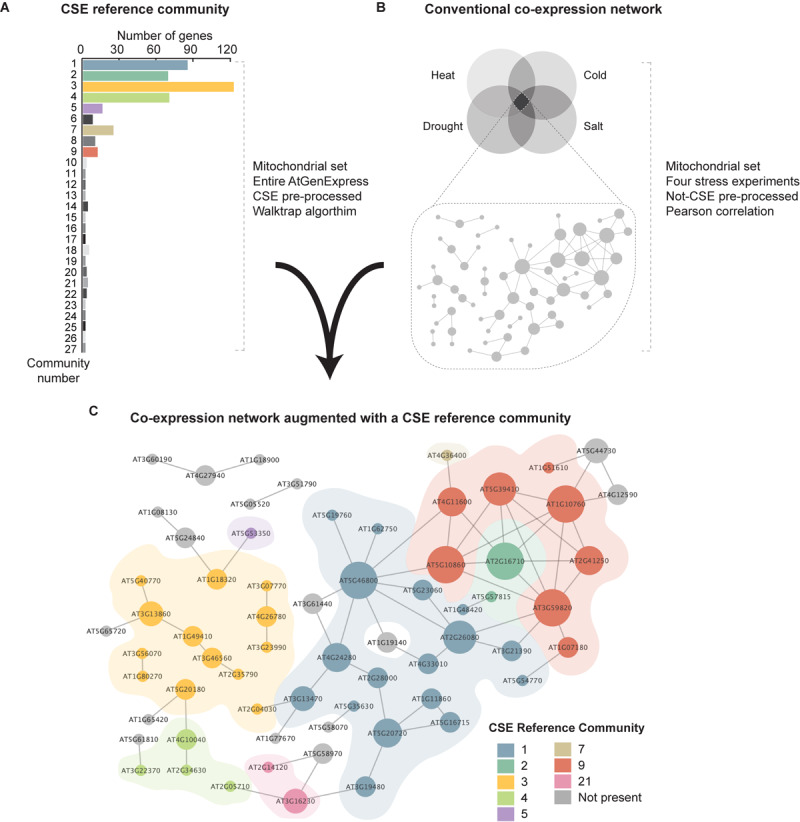
Synthesis of a conventional co-expression network of Arabidopsis shoots common to four stresses with a CSE reference community. **(A)** A CSE reference community was generated utilizing the entire AtGenExpress Expression Atlas (Schmid et al., 2005; Kilian et al., 2007; Goda et al., 2008), using CSE pre-processed data. This network was divided into 27 primary clusters using a Walktrap community detection algorithm (Pons and Latapy, 2005). **(B)** A core set of stress-responsive genes was isolated from the AtGenExpress stress dataset (Kilian et al., 2007) covering heat, drought, cold, and salt stresses and from this, a network was generated based on Pearson correlation coefficient with no CSE. **(C)** The initial network of non-CSE core stress response generated using Pearson correlation coefficient was cross-referenced with the CSE reference community; providing deeper insight into the connectivity between genes, independent of outside influences such as stress or tissue type. The diameter of each node is proportional to the number of edges it has to a neighboring node and node coloration denotes occupation within a given CSE reference community.

In the field of transcriptomics, the application of conventional co-expression networks has proven a highly powerful approach in characterizing stress responses in a diversity of organisms. In this study, we have demonstrated that CSE of data prior to correlation analysis effectively identifies the innate relationship between genes, and thus delineates a “core gene-network”. However, as previously mentioned, a caveat of this approach is that it is predicated on the suppression of extraneous effects, such as stress, tissue, treatment, or genotype from a given dataset, which therefore hinders downstream efforts to interrogate the impact of these outside influences on the dynamics of the resulting GCN. On the other hand, quite often researchers must adjust different parameters (cut-offs, thresholds, etc.) to introduce enough genes to reposition the stress-responsive network in a wider biological context and gain understanding. Here, we propose an alternative method, with a powerful reference tool that can augment conventional co-expression analyses. By clustering the CSE data of the entire AtGenExpress Expression Atlas using a Walktrap community detection algorithm (Pons and Latapy, 2005), we generated a hierarchical CSE reference community composed of 27 communities (Figure 6A). This additional filter based on co-expression metadata could then be layered onto a conventional, i.e. non-CSE GCN (based on any treatment, developmental stage, or tissue type selected by the researcher), and thus provide a more detailed and nuanced view of the innate relationships between the genes, when stress/treatment/tissue/genotype effects have been nullified.

To illustrate this, we identified a subset of 65 mitochondrial genes that are highly co-expressed in shoot tissues in response to the following four stress treatments: heat, cold, drought, and salt, using non-CSE pre-processed data (Kilian et al., 2007). As shown in Figure 6B, conventional co-expression analysis (here based on Pearson correlation coefficient) provides an initial network, which illustrates the influence of various stresses on the relationship between specific stress-responsive genes. When the expression network of the core stress responsive genes was cross-referenced with the CSE reference community, the resulting subdivisions revealed unique insights into the functional composition and basal connectivity of this network (Figure 6C and Supplementary Table S5). For example, most of genes grouped in Community 1 were associated with photorespiration and thiamine biosynthesis, two metabolic pathways often associated with stress response in plants, and notably in photosynthetic tissues (Supplementary Figure S4) (Rapala-Kozik et al., 2012; Hodges et al., 2016). Furthermore, Community 3 was overwhelmingly composed of functions associated with translation (e.g. ribosomal protein L36), import (e.g. TOM6, TIM9, and the TIM-family protein AT1G18320), and assembly (e.g. HSP60-3A, HSP6, Hsp89.1, CR88, and MGE2). Interestingly, a number of the genes in this core stress set prepared from shoot samples were also present in a corresponding network prepared from root data (denoted with a black outline in Figure 6B). Of these shared genes, 2/3rd are found in Community 3, which again emphasizes their importance. Therefore, we propose that viewing traditional GCNs through a prism of a CSE reference community can rapidly reveal hidden degrees of connectivity between genes and could have far-reaching applications in the field of transcriptomics, regardless of organisms, treatments or pathologies.

## Discussion

In light of the burgeoning output of next generation sequencing projects performed on a variety of species, tissues, developmental or clinical conditions, the statistical power and complexity of these networks will undoubtedly increase, while their biological relevance will be fiercely challenged. Therefore, it is essential that current methodologies be refined to keep apace of this progress and utilize these resources to generate more accurate and informative gene networks to answer hypothesis-driven questions. With the present study, we proposed an alternative method to conventional batch corrections and demonstrated that the implementation of CSE (performed simultaneously per gene and per sub-experiment) to conventional correlation approaches can provide additional biological relevance to GCNs.

Arguably, there is no universal GCN that can define the relationship between genes under every conceivable tissue, developmental stage or treatment. Nonetheless, we believe there is utility in approximating this by generating a core network, where the edges correspond to essential interactions and highlight conserved pathways. Furthermore, the predicted number of edges in a GCN is user-defined, e.g. an edge is predicted if the correlation is significant and/or has a value greater than an arbitrary threshold. From a biological point of view, these inclusion criteria are problematic since the number of edges depends on (i) the number of samples (the more samples, the lower the *P*-values and thus the more edges) and (ii) which method is used to calculate co-expression. For example, GCNs using CSE will on average estimate fewer extreme correlations than GCNs not using CSE, although they may share several edges (Table 1 and Supplementary Figure S5). We argue that a sensible alternative approach is to control the sparsity of the network and to consider the predicted edges simply as the most pronounced co-expression.

**TABLE 1:** Table of pros and cons associated with implementing CSE approaches in GCN analysis.

	**Cons**	**Pros**
**Non-CSE**	• Struggles with complex heterogenous dataset, i.e. ranging from different treatments/tissues samples • Prone to generating false positives, i.e. co-expression confounded by external factors	• Allows the user to process homogeneous datasets • Retains and queries conditional effects such as treatment/stress • Well established methods
**CSE**	• Large dataset required • Limited detection of co-expression driven by external factors	• Facilitates analyses of complex heterogenous dataset originating from different time points/tissues/treatments/stresses • Provides a “core network”, which can act as a reference for comparison analyses • Effectively reduce the amount of false positives • Easy to implement to any pre-existing workflow

The predicted core network depends on the coverage of included samples, which necessitates extensive sampling; covering different tissue types, developmental stages, and stresses. Yet, a consequence of sampling broadly is the integration of samples from contextually different experiments, with core gene co-expression being obscured by treatment-associated co-expression. One interesting solution would be to split the experimental data into subsets where each subset consists of data from similar experiments, and predict a separate network for each dataset, and finally estimate the core network with a consensus network. However, such approach would still suffer from some shortcomings; it may be difficult to define the subsets, there may be relatively few samples within the subsets and it is unclear how to derive the consensus network. To a certain extent, a CSE-based network can be regarded as an extreme consensus network, which bases the analysis on the smallest homogenous subsets and use all CSE-processed samples to estimate the GCN. The proposed CSE pre-processing method, which can theoretically be combined with any GCN method, defines the subsets (i.e. the sub-experiments) conservatively and mechanically, where each sub-experiment consists of biological replicates, and removes all treatment effects including batch effects, thus allowing for a direct estimation of the core network based on all available samples. A drawback with the CSE approach is that it will reduce the signal-to-noise ratio (Table 1). For the considered Arabidopsis data, with 887 samples, this seems to be a minor problem, but for relatively small data sets it remains an open question whether this could become a hurdle. Recently Kuijjer et al. (2019) proposed a novel approach to derive sample-specific regulatory networks from an estimated GCN. An interesting, but as yet unexplored idea, would then be to base the sample-specific regulatory networks on a CSE-derived GCN and then use the sample-specific networks to predict tissue/stress specific networks.

Evaluation and validation of GCNs is a challenge, since we have limited information on the “true” relationship that exists between genes. We commonly have experimentally confirmed protein–protein interactions and for some subsets of genes it may be reasonable to assume a relatively high degree of co-expression. We usually lack information on truly non-existing edges. In fact, from a theoretical point of view, we may argue that all pairs of genes are co-expressed to some extent. We propose that the validation should be based on pronounced sub-networks for which we expect to observe higher co-expression (i.e. more edges) than expected by chance. This approach allows us to compare different GCNs, all with the same sparsity, and to easily assess statistical significance. It should be stressed that the result of the validation depends on the sparsity level and which pronounced sub-networks are used in this validation (Table 1). In particular, if the number of genes is high it may be recommended to construct a relatively dense network and to include several pronounced sub-networks to ensure high power of the tests.

In this study, we used a plant mitochondrial case study, where a series of validation steps established the strength of GCNs built upon data that had been pre-processed with CSE. Plant mitochondria are highly adaptive organelles that can tailor their protein complement to undertake a multitude of specialized roles. Nonetheless, there are a set of canonical functions and associated pathways that are maintained in most tissues, growth conditions, developmental stage, etc. even though such pathways (e.g. respiration, TCA cycle, amino acid catabolism) can of course be differentially regulated to modulate activity i.e. regulation of metabolic fluxes. This means that the genes encoding proteins involved in those pathways are functionally correlated even though their respective expression profiles may diverge slightly to satisfy a certain metabolic modularity. Our results show that CSE-based conventional GCNs (Pearson, partial, WGCNA) had significantly more edges within the majority of the considered pronounced sub-networks (i.e. the mETC and its complexes; and sub-networks defined by functional annotation) than GCNs not using CSE (Figures 3, 4 and Supplementary Figures S2, S3); which demonstrates that the CSE-based GCNs are efficient at predicting canonical functions and associated pathways. Furthermore, we showed that CSE, in conjunction with Pearson correlation can be used to fine-tune the prediction of the function of uncharacterized genes (Figure 5); while combination with non-CSE data can augment conventional stress analyses with the innate connections underpinning the dynamic system examined (Figure 6). Furthermore, we benchmarked our method to existing pipelines for gene network analyses and demonstrated that CSE in combination with either Pearson or partial correlations was significantly superior overall (Supplementary Table S6). Three of the tested methods are included in the NetMiner pipeline (Yu et al., 2018), which was developed to make a consensus GCN from RNA-seq data. This method uses a voting system to generate the consensus pipeline based on WGCNA, BC3Net, and GeneNet methods. As our dataset was based on microarray data, which estimates transcript abundance based on fluorescence rather than read counts, we have not directly used NetMiner but instead compared BC3Net and GeneNet, in addition to the four methods already employed in our study. While CSE does not enhance the biological relevance of BC3Net and GeneNet approaches, it demonstratably improves the depth of the WGCNA approach. Yet, at this stage, it remains unclear why BC3Net and GeneNet approaches were not affected by centralization. Nonetheless, these two methods arguably performed worse than the more conventional approach of Pearson correlation with centralized data. Of note, in our study, GeneNet does not seem to provide relevant data as the number of edges is equal to what is predicted by chance, which thus seems biologically unsound.

Indeed, the trade-off of implementing a CSE approach is that the biological precision gained by strengthening a core gene-network results in a loss of information from any stress/treatment/genotype components of the dataset (Table 1). Despite this, if the focus of a given study is centered on determining the network articulated around specific stress-responsive genes, one can apply a CSE reference community onto a conventional “stress” co-expression network. This augments the network with extended biological insights, and provides the user with a resource to better interrogate the biological context of the data. Such context is often hindered by the use of stringent cut-offs and thresholds throughout GCN establishment (Table 1). Finally, although based on a plant mitochondrial set to streamline the biological validation of our method, the present study provides an alternative approach for interrogating the biological relevance of any GCN, regardless of organism or biological context.

## Materials and Methods

### Dataset Generation

To obtain the widest coverage possible of a plant transcriptome, the AtGenExpress expression atlas was utilized. This resource is the result of a multinational consortium that aimed to define an exhaustive transcriptome, covering (i) Arabidopsis developmental stages and tissues types (Schmid et al., 2005), (ii) biotic and abiotic stress treatments (Kilian et al., 2007), and (iii) hormone and chemical treatments (Goda et al., 2008). These studies used Affymetrix ATH1 arrays and, where possible, maintained consistent experimental practices between samples so as to optimize comparability. For this study, 887 CEL files from the AtGenExpress set (spanning over 370 unique experimental conditions) were quantile normalized together resulting in the pre-processed dataset. For each unique condition (henceforth referred to as sub-experiment) there were two or three samples, which can be regarded as biological replicates observed under similar conditions, where the conditions were defined with respect to tissue developmental stage and treatment, e.g. a different type of stress (see Supplementary Table S1).

### Construction of Gene Co-expression Networks

All analysis, if not stated elsewhere, was conducted with the statistical programming language R version (R 3.5.1) (R Core Team, 2018). The R-code used to construct the GCNs described below are found in our GitHub repository^1^ (Kellgren and Rydén, 2019).

Pearson correlation was obtained using the function “cor” in R and the partial correlation was obtained using the function “pcor” with default setting in the R-package “ppcor” (Kim, 2015).

The adjacency matrices were derived by controlling the fraction of edges in the off-diagonal adjacency matrix at a user defined level ω. The elements of the adjacency matrix were derived from a correlation matrix where the elements were set to “1” if the absolute value of the correlations were larger than a cut-off α, and “0” otherwise. The threshold α was obtained by an iterative procedure controlling the sparsity at the level ω = 0.005.

The above approach was used for all analyses with the exception of the analysis resulting in the predicted reference communities presented in Figure 6, where an alternative bootstrap approach was used. Here, samples were randomly chosen with replacement, followed by calculation of the adjacency matrix as described above. This procedure was repeated 50 times and the resulting adjacency matrices were combined, generating a matrix with values ranging from 0 to 50. The elements of the adjacency matrix were derived from the aggregated matrix, where the elements were set to “1” if the values exceeded a cut-off β, and “0” otherwise. Here β was chosen to control the sparsity ω at 0.005.

Due to computational constraints, partial correlation approaches are often carried out on subsets of genes, rather than the whole genome of an organism. An example of this was detailed in Ma et al. (2007), which used a modified GCN approach to carry out partial correlation analysis on batches of ∼2000 genes at a time. Aided by iterative random samplings of genes, this study increased their coverage to that of the Affymetrix ATH1 array; resulting in a network composed of 18,625 interactions (edges) and 6760 genes (nodes) (Ma et al., 2007). Ren et al. (2015) expanded on this and proposed an algorithm for constructing GCN with high-dimensional data by implementing asymptotically normal estimation of large GCNs, and in doing so, made it realistic to perform partial correlation at a whole-genome scale (Wang et al., 2016). Unsurprisingly, this approach is enormously computationally taxing, which can prove prohibitive to researchers lacking dedicated servers and advanced computer processing power.

The WGCNA networks were prepared using unsigned biweight midcorrelation (Song, 2012) with a soft thresholding power of 5 as the weight function. A topological overlap metric (TOM) similarity matrix (Yip and Horvath, 2007) was derived from the resulting correlation matrix that in turn was used to derive an adjacency matrix with a sparsity of 0.005. The WCGNA Consensus networks were derived by splitting the original data into five subsets based on tissue (flower *n* = 63, leaf = 168, root *n* = 133, shoot *n* = 154, and seedling *n* = 207) while the remaining 162 samples were removed from the analysis. For each subset an unsigned biweight midcorrelation network with a soft thresholding power of 6 (CSE data) or 11 (non-CSE data) was constructed and used to construct a TOM similarity matrix. A consensus TOM similarity matrix was derived by combining the five tissue-specific TOM matrices by, for each cell, taking the minimum value the five TOM matrices. The consensus TOM was then converted to an adjacency matrix with a sparsity of 0.005. The analysis was carried out using the R package WGCNA v 1.68 (Langfelder and Horvath, 2008, Langfelder and Horvath, 2012).

The BC3Net networks were constructed by using the function “bc3net” with default settings except increasing to 200 bootstrap datasets and the igraph parameter to FALSE in the R “bc3net” package (de Matos Simoes and Emmert-Streib, 2016). The “ggm.estimate.pcor” function from the R package “GeneNet” (Schäfer et al., 2020) with default settings was used to construct the GeneNet networks.

### Evaluation of Gene Co-expression Networks

We consider a predicted network with sparsity ω. For any sub-network, with *n* nodes and *K* observed edges it is possible to test if the sub-network is pronounced (i.e. the sub-network has significantly more edges than expected by chance) versus that the sub-network is not pronounced (the null hypothesis). Under the null hypothesis *K* is binomial distributed, i.e.

$$K∼B\,i\,n\,\left(\left(\begin{array}{c} n\\ 2 \end{array}\right),\omega \right)$$

Here, the binomial test, using the R-function “binom.test” with a one-sided alternative hypothesis, was used to derive the *p*-values of interest. It should be stressed that the *P*-values depend on the sparsity. Hence, all tough not necessary, having the same sparsity in all networks simplifies the evaluation. In addition to test if the sub-networks are pronounced it is also of interest to compare two predicted networks, e.g. network *A* and *B*. We consider a sub-set with n nodes, where we observe *K*_*A*_ and *K*_*B*_ edges within the sub-network for network *A* and *B* respectively. Here the null hypothesis is that the expected values of *K*_*A*_ and *K*_*B*_ are the same and the alternative hypothesis that they differ. Fisher’s exact test, using the R-function “fisher.test” (R 3.5.1) with a two-sided alternative hypothesis, was used to derive the *P*-values of interest. For some selected genes we tested if the gene had more (or less) edges then expected by chance to genes within a functional category. The binomial test, using the R-function “binom.test” with a one-sided alternative hypothesis, was used to test the hypothesis.

### *Z*-Score Analysis

*Z*-score analysis was carried out to compare two proportions (subset versus whole mitochondrial set) to determine if there was a statistically significant overrepresentation or underrepresentation of a particular MapMan subcategory. In the calculation below, π refers to the mean and *n* is the number of genes in the subset.

$$z=\frac{\hat{\pi_{1}}-\hat{\pi_{2}}}{\sqrt{\hat{\pi }\,\left(1-\hat{\pi }\right)\,\left(\frac{1}{n_{1}}+\frac{1}{n_{2}}\right)}}$$

Following this calculation, a cumulative standard normal table was used to match the *z*-score and determine the *P*-value.

### Preparing Elements of the Mitochondrial Working Model

#### (i) Defining the Mitochondrial Gene List

The manually curated list of genes encoding proteins targeted to the mitochondrion from Chrobok et al. (2016) was used as a basis for a mitochondrial case-study. Matching this list with the AtGenExpress Expression Atlas resulted in a list of 984 mitochondrial genes, which were used for downstream analysis. The samples were taken from different tissues: flower, root, shoot, seedling, leaf, pollen, and silique. Mitochondrial genes were categorized with respect to expression patterns, functional proximity and functional categories for downstream validation (Supplementary Table S1).

#### (ii) Defining Below-Ground and Above-Ground Dominant Genes

The mitochondrial genes were classified into two categories with respect to their expression patterns in below-ground tissues (e.g. root) and above-ground tissues (e.g. shoot and leaf). For each gene *i*, the difference between the mean expressions in below-ground tissues, ${\bar{x}}_{B\,i}$ and above-ground tissues, ${\bar{x}}_{A\,i}$ was calculated, i.e. $\Delta_{i}={\bar{x}}_{B\,i}-{\bar{x}}_{A\,i}$. Genes with a difference larger than one standard deviation, i.e. Δ_*i*_ > *s*_*Δ*_, were classified as *below-ground dominant genes*, while those with a difference smaller than one standard deviation, i.e. Δ_*i*_ < −*s*_Δ_, were classified as *above-ground dominant genes.* The estimated standard deviation was based on all the Δ-values of genes.

#### (iii) Defining Components of Complex I of the Mitochondrial Electron Transport Chain

Complex I of the mitochondrial electron transport chain (mETC) was an ideal model to test the effect of functional proximity of the resulting networks, as the identity and molecular arrangement of these constituents have been thoroughly characterized in Arabidopsis using proteomic approaches (Klodmann et al., 2010; Peters et al., 2013).

#### (iv) MapMan Annotations

Using the newly updated functional annotations established for the MapMan platform (MapMan X4 Release 1.0, 2018; Usadel et al., 2009), each gene of the mitochondrial set was assigned to one of 29 functional categories.

### Preparing a Reference Community Set

The *Walktrap community detection algorithm* runs short random walks and merges separate communities in a bottom-up manner to produce clusters, and was applied to the derived networks to identify *gene communities*, i.e. sets of genes with a high degree of predicted intra-gene-gene interactions. The function “walktrap.community” with default settings in the R package igraph (Csárdi and Nepusz, 2006) was used to conduct the analyses. Here, gene communities were predicted based on a network obtained using CSE data from all experiments, Pearson correlation and an adjacency matrix derived using the absolute value of the correlations. The result was a CSE reference community composed of 27 clusters.

### Combining Results Obtained Using CSE and Non-CSE Data

We claim that gene communities should be estimated based on networks derived using all the available CSE data, while networks based on non-CSE data describe how genes are affected by an external factor, e.g. stress induced by heat, cold, salt or drought. Combining the two type of networks allowed us to study how gene communities were affected by stress.

The combined analysis was made as follows. First the communities were predicted as described above, resulting in the *community network*. Secondly, for each of the considered stresses, samples exposed to the stress were selected (heat *n* = 16, cold *n* = 24, salt *n* = 24, and drought *n* = 28). An adjacency matrix was calculated using non-CSE data, Pearson correlation, and non-bootstrap approach with a cut-off = 0.82. The sum of the four stress-related adjacency matrices was calculated and edges with an aggregated score equal to 4 were set to “one” in the combined adjacency matrix (i.e. the *stress network*) and regarded as gene-gene interaction caused by a general stress response.

The community and stress networks were combined. Communities enriched with respect to general stress were identified similarly as described above. An enrichment analysis with respect to functional categories was made for each of the enriched communities.

## Data Availability Statement

All datasets generated are included in Supplementary Data and at the following location: https://www.upsc.se/researchers/4638-olivier-keech-stress-induced-senescence-and-its-subsequentmetabolic-regulations.html#resources, https://github.com/Tezinha/Gene-Co-expression-Network.

The datasets analyzed during the current study are available under the AtGenExpress expression atlas, which is the result of a multinational consortium that aimed to define an exhaustive transcriptome, covering (i) Arabidopsis developmental stages and tissues types (Schmid et al., 2005), (ii) biotic and abiotic stress treatments (Kilian et al., 2007), and (iii) hormone and chemical treatments (Goda et al., 2008).

## Author Contributions

SL, TK, and RB performed analyses. SL prepared the figures and drafted the manuscript. PR and OK conceptualized the project and edited the manuscript. All authors read and approved the manuscript.

## Conflict of Interest

The authors declare that the research was conducted in the absence of any commercial or financial relationships that could be construed as a potential conflict of interest.

## Footnotes

1 https://github.com/Tezinha/Gene-Co-expression-Network

## Supplementary Material

FIGURE S1 Schematic representations of the conclusions that can be drawn from different correlation analysis approaches of gene expression data. Five genes were simulated to illustrate a network in the following way; Gene A expression affects Gene B expression, Gene C expression affects the expression of Gene D and Gene E. The gene’s expression values are regarded as functions of a normally distributed random variable, with a mean μ = 0, and a standard deviation σ = 0.5. The expression of two of the genes, Gene A and Gene C are also affected by an external stress treatment, which can be seen as a categorical variable with two levels. Level one represents no external influences and the variable takes a value of zero, at level two the gene is influenced by an external factor and the categorical variable takes the value ten. Gene B expression is affected by the expression of Gene A, so for each Gene B value a Gene A value multiplied by a constant β = 0.5 is added. In the same way, Gene D and Gene E is simulated but with the exception that they are affected by Gene C. For each of the scenarios 100 expression values were simulated for each gene. To compare Pearson’s correlation against partial correlation the relative correlation, i.e. the most correlated edge, was set as a baseline and received a correlation value of 1. This was done for each setup. In the first column the true network is represented and if it is affected by the external factor. In column 2 to 5 the strength of the relative correlations is represented by the thickness of the line. **(A)** The network is not affected by any external factor and all four methods have the correct edges among the top three candidates. There is no difference between non-CSE and CSE data which is as expected when there is no external factor to remove with CSE. **(B)** The stress treatment is affecting gene C expression, which has an effect on the non-CSE networks. Pearson correlation gives a false positive among the top three candidates, the partial correlation networks gives the correct top three candidates but the edge between Gene A and B is weak. When we preform CSE both networks give the correct top three edges. **(C)** In this case, the stress treatment is affecting the expression of both Gene A and C, which leads to false positives with both methods. By carrying out CSE, the stress treatment, is removed and both Pearson and partial correlation output the correct top three edges.

FIGURE S2 Comparative analysis of four different correlation methods in defining interactions based on functional proximity. The following gene subsets of the mitochondrial electron transport chain were analyzed using non-CSE WGCNA All, non-CSE WGCNA Consensus, CSE WGCNA All, and CSE WGCNA Consensus. *P*-values were calculated (two-tailed binomial test) for the probability associated with the expected vs. observed number of edges and a color-grading scheme of the resulting *P*-values applied. **(A)** A Venn diagram illustrating the overlap of connections between the complexes of the mitochondrial electron transport chain (mETC), when analyzed using the four different correlation methods. Two tailed Fisher’s exact test was used to test the significance of the number of edges within the mETC between CSE and non-CSE approaches. **(B)** The significance of the edges between the three domains of Complex I. **(C)** The significance of the edges within a given complex or between the different complexes of the ETC. **(D)** Between the individual complexes of the mETC vs. the unified mETC or the rest of the mitochondrial set excluding the mETC.

FIGURE S3 Comparative analysis of four different correlation methods based on connectivity between different functional categories in mitochondria. Using newly updated MapMan annotations (MapMan X4 Release 1.0, 2018; Usadel et al., 2009), the mitochondrial set was subdivided into 29 different functional categories. Only functional categories with at least one significant (*P* < 0.0001; one-tailed binomial test) connection to another category are displayed for each method. Nodes with a black outline indicate functional categories with significant intra-connectivity, nodes lacking an outline indicates functional categories that do not have a significant (*P* < 0.001) number of edges within a functional category. Lines between functional categories indicate a significant (*P* < 0.0001) number of edges exist between the genes comprising both function categories. The presence of an ^∗^ indicates that based on a two-tailed Fisher’s exact test, there is a significantly (*P* < 0.05) greater proportion of edges when comparing CSE-processed data vs. non-CSE data**. (A)** Non-CSE WGCNA All, **(B)** non-CSE WGCNA Consensus, **(C)** CSE WGCNA All, and **(D)** CSE WGCNA Consensus.

FIGURE S4 Synthesis of a conventional co-expression network of Arabidopsis shoots common to four stresses with a CSE Reference Community Set. A core set of stress-responsive genes isolated from non-CSE AtGenExpress stress dataset (Kilian et al., 2007) covering heat, drought, cold, and salt stresses, cross-referenced with the CSE Reference Community.

FIGURE S5 Correlation between the 985 mitochondrion related genes were estimated using Pearson correlation without centralization (Non-Centralized data) and Pearson correlation with CSE preprocessing (CSE preprocessed data). For each approach 484,620 correlations were estimated and the 0.5% (2423) gene correlations with the highest absolute value were used to predict edges in the corresponding gene co-expression network. **(A)** Estimated density functions over all estimated correlations for non-centralized data (green) and CSE preprocessed data (red). The black line shows the density for correlations estimated on simulated noise. **(B)** The estimated correlations for the two approaches plotted against each other. Edges shared by both approaches are marked blue (620 (25.6%) of the edges were shared), unique edges for the CSE preprocessing network are marked red, and unique edges for the Non-centralized network are marked green.

TABLE S1 List of 984 genes encoding proteins targeted to the mitochondrion, referenced with the AtGenExpress Expression Atlas (Schmid et al., 2005; Kilian et al., 2007; Goda et al., 2008). Note that dues to its large size (ca. 250 MB), the file is available at: https://www.upsc.se/documents/olivier/Supplemental_Table_1_Law_et_al_2020_DOI_10.3389-fpls_2020:00524.xlsx

TABLE S2 **(i)** Non-CSE Pearson correlation; **(ii)** non-CSE partial correlation; **(iii)** CSE Pearson correlation; **(iv)** CSE partial correlation.

TABLE S3 Statistics supporting Figure 3. Table of the expected, observed, ratios, and associated *P*-values. This is carried out for interactions within Complex I, within and between the five Complexes of the mETC, and between the mETC and the rest of the mitochondrion.

TABLE S4 List of source and target genes comprising genes encoding proteins targeted to the mitochondrion, with unknown functions (as per MapMan X4 annotations) and their edges with known mitochondrial genes.

TABLE S5 Table of the 27 communities generated using the Walktrap algorithm on the whole AtGenExpress Set that has been centralized.

TABLE S6 Comparison of the number of edges observed within the mETC and within the functional categories (WFC) 1-27 using different approaches, with or without CSE. Six methods were included: Pearson correlation, partial correlation, WGCNA, WGCNA Consensus network, BC3Net, and GeneNet; and these were utilized on either non-centralized data (non-CSE) or centralized data (CSE). All networks were fixed to a similar sparsity, each with approximately 2400 edges (0.5%). The number of edges observed for the different approaches and data were compared to the number of edges expected by chance within the mETC and WTC. Here, blue numerals indicate that the observed number was significantly larger (*p* < 0.05) than expected by chance. For each approach, the performance applying centralized versus non-centralized data were tested. Here, an ^∗^ indicates that significantly more edges (*p* < 0.05) were observed when using CSE adjusted data. Finally, the results obtained using Pearson correlation and centralized data (PeCSE) were compared to all other methods. Approaches with significantly fewer (*p* < 0.05) number of edges than PeCSE were marked with a minus sign ‘−’.
