# Supplementary material for: Centralization Within Sub-Experiments Enhances the Biological Relevance of Gene Co-expression Networks: A Plant Mitochondrial Case Study
Source: Front Plant Sci. 2020 Jun 4;11:524. doi: 10.3389/fpls.2020.00524 (PMC7287149; doi:10.3389/fpls.2020.00524)
Supplement: FIGURE S1 — Schematic representations of the conclusions that can be drawn from different correlation analysis approaches of gene expression data. Five genes were simulated to illustrate a network in the following way; Gene A expression affects Gene B expression, Gene C expression affects the expression of Gene D and Gene E. The gene’s expression values are regarded as functions of a normally distributed random variable, with a mean μ = 0, and a standard deviation σ = 0.5. The expression of two of the genes, Gene A and Gene C are also affected by an external stress treatment, which can be seen as a categorical variable with two levels. Level one represents no external influences and the variable takes a value of zero, at level two the gene is influenced by an external factor and the categorical variable takes the value ten. Gene B expression is affected by the expression of Gene A, so for each Gene B value a Gene A value multiplied by a constant β = 0.5 is added. In the same way, Gene D and Gene E is simulated but with the exception that they are affected by Gene C. For each of the scenarios 100 expression values were simulated for each gene. To compare Pearson’s correlation against partial correlation the relative correlation, i.e. the most correlated edge, was set as a baseline and received a correlation value of 1. This was done for each setup. In the first column the true network is represented and if it is affected by the external factor. In column 2 to 5 the strength of the relative correlations is represented by the thickness of the line. (A) The network is not affected by any external factor and all four methods have the correct edges among the top three candidates. There is no difference between non-CSE and CSE data which is as expected when there is no external factor to remove with CSE. (B) The stress treatment is affecting gene C expression, which has an effect on the non-CSE networks. Pearson correlation gives a false positive among the top three candidates, the pa [file Image_1.pdf]

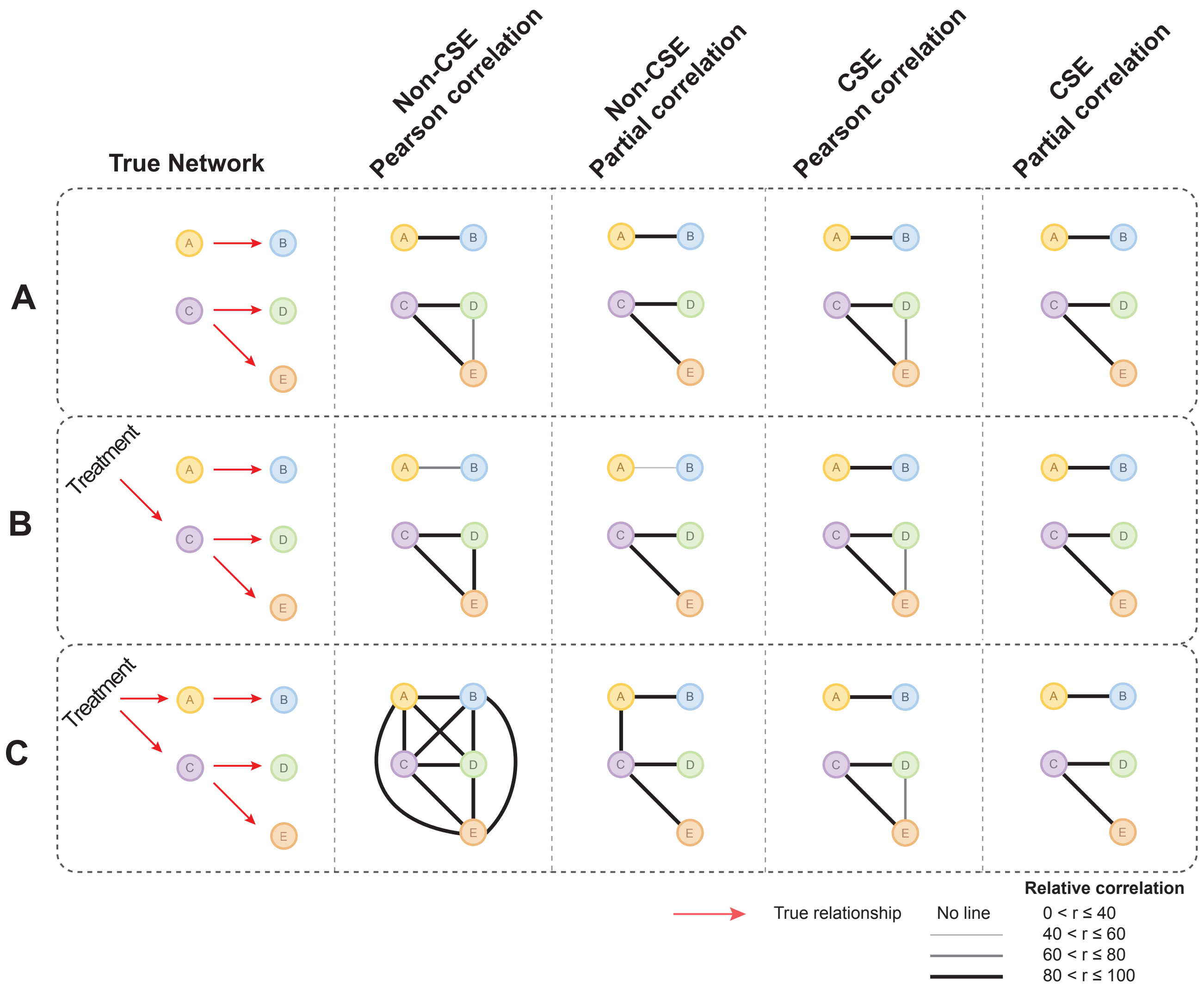

Supplemental Figure 1. Schematic representations of the conclusions that can be drawn from different correlation analysis approaches of gene expression data. Five genes were simulated to illustrate a network in the following way; Gene A expression affects Gene B expression, Gene C expression affects the expression of Gene D and Gene E. The gene's expression values are regarded as functions of a normally distributed random variable, with a mean μ=0, and a standard deviation σ=0.5. The expression of two of the genes, Gene A and Gene C are also affected by an external stress treatment, which can be seen as a categorical variable with two levels. Level one represent no external influences and the variable takes a value of zero, at level two the gene is influenced by an external factor and the categorical variable takes the value ten. Gene B expression is affected by the expression of Gene A, so for each Gene B value a Gene A value multiplied by a constant β=0.5 is added. In the same way, Gene D and Gene E is simulated but with the exception that they are affected by Gene C. For each of the scenarios 100 expression values were simulated for each gene. To compare Pearson's correlation against partial correlation the relative correlation, i.e. the most correlated edge, was set as a baseline and received a correlation value of 1. This was done for each setup. In the first column the true network is represented and if it is affected by the external factor. In column 2 to 5 the strength of the relative correlations is represented by the thickness of the line. **(A)** The network is not affected by any external factor and all four methods have the correct edges among the top three candidates. There is no difference between non-CSE and CSE data which is as expected when there is no external factor to be remove. **(B)** The stress treatment is affecting gene C expression, which has an effect on the non-CSE networks. Pearson correlation gives a false positive among the top three candidates, the partial correlation networks give the correct top three candidates but the edge between Gene A and B is weak. When we preform CSE both networks give the correct top three edges. **(C)** In this case, the stress treatment is affecting the expression of both Gene A and C, which leads to false positives with both methods. By carrying out CSE, the stress treatment, is removed and both Pearson and partial correlation output the correct top three edges.
