# Supplementary material for: Centralization Within Sub-Experiments Enhances the Biological Relevance of Gene Co-expression Networks: A Plant Mitochondrial Case Study
Source: Front Plant Sci. 2020 Jun 4;11:524. doi: 10.3389/fpls.2020.00524 (PMC7287149; doi:10.3389/fpls.2020.00524)
Supplement: FIGURE S2 — Comparative analysis of four different correlation methods in defining interactions based on functional proximity. The following gene subsets of the mitochondrial electron transport chain were analyzed using non-CSE WGCNA All, non-CSE WGCNA Consensus, CSE WGCNA All, and CSE WGCNA Consensus. P-values were calculated (two-tailed binomial test) for the probability associated with the expected vs. observed number of edges and a color-grading scheme of the resulting P-values applied. (A) A Venn diagram illustrating the overlap of connections between the complexes of the mitochondrial electron transport chain (mETC), when analyzed using the four different correlation methods. Two tailed Fisher’s exact test was used to test the significance of the number of edges within the mETC between CSE and non-CSE approaches. (B) The significance of the edges between the three domains of Complex I. (C) The significance of the edges within a given complex or between the different complexes of the ETC. (D) Between the individual complexes of the mETC vs. the unified mETC or the rest of the mitochondrial set excluding the mETC. [file Image_2.pdf]

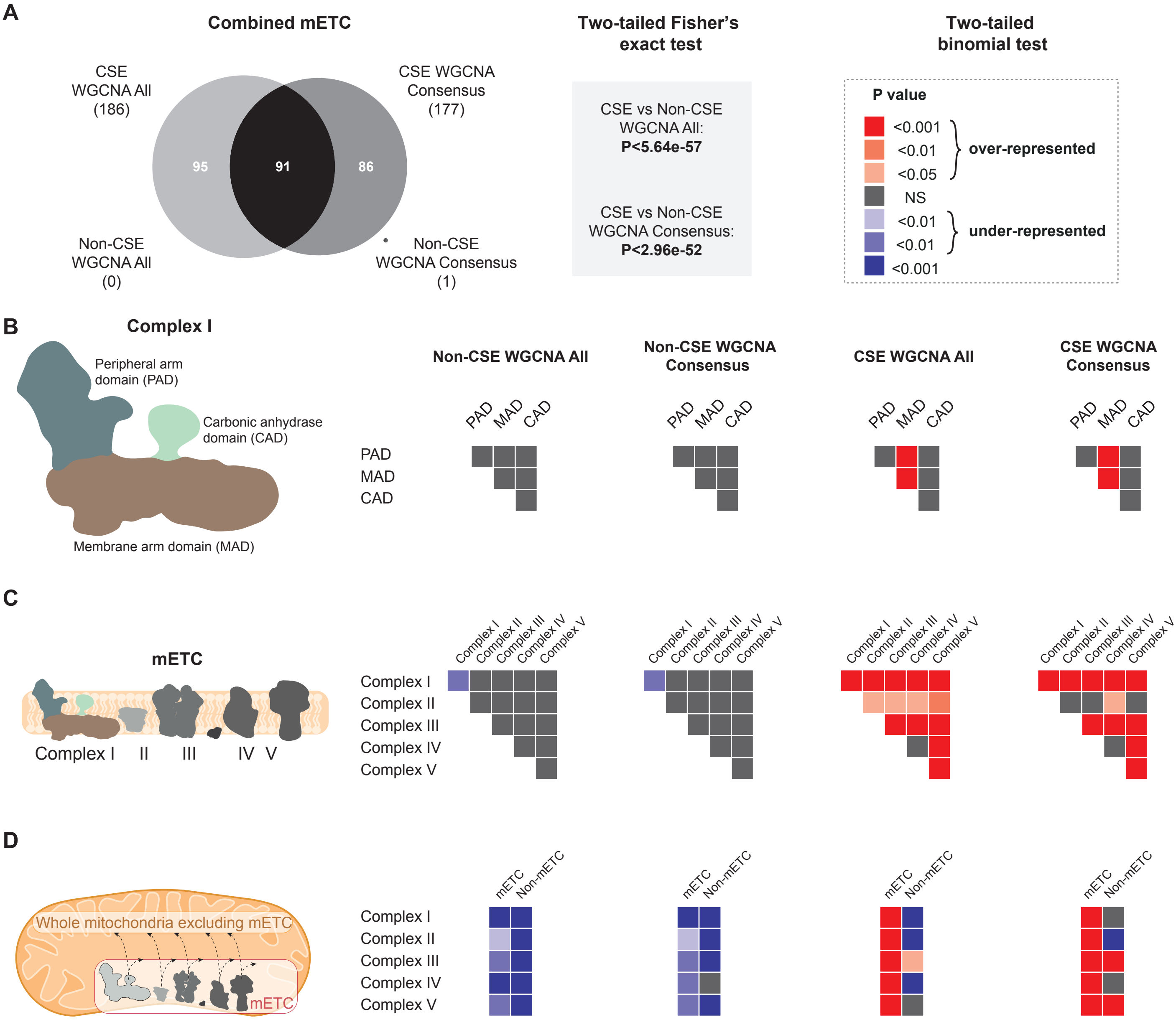

Supplemental Figure 2. Comparative analysis of four different correlation methods in defining interactions based on functional proximity. The following gene subsets of the mitochondrial electron transport chain were analysed using Non-CSE WGCNA All, Non-CSE WGCNA Consensus, CSE WGCNA All and CSE WGCNA Consensus. P values were calculated (two-tailed binomial test) for the probability associated with the expected vs. observed number of edges and a colour-grading scheme of the resulting P values applied. **(A)** A Venn diagram illustrating the overlap of connections between the complexes of the mitochondrial electron transport chain (mETC), when analysed using the four different correlation methods. Two tailed Fisher's exact test was used to test the significance of the number of edges within the mETC between CSE and Non-CSE approaches. **(B)** The significance of the edges between the three domains of Complex I. **(C)** The significance of the edges within a given complex or between the different complexes of the ETC. **(D)** Between the individual complexes of the mETC vs the unified mETC or the rest of the mitochondrial set excluding the mETC.
