# Supplementary material for: Centralization Within Sub-Experiments Enhances the Biological Relevance of Gene Co-expression Networks: A Plant Mitochondrial Case Study
Source: Front Plant Sci. 2020 Jun 4;11:524. doi: 10.3389/fpls.2020.00524 (PMC7287149; doi:10.3389/fpls.2020.00524)
Supplement: FIGURE S3 — Comparative analysis of four different correlation methods based on connectivity between different functional categories in mitochondria. Using newly updated MapMan annotations (MapMan X4 Release 1.0, 2018; Usadel et al., 2009), the mitochondrial set was subdivided into 29 different functional categories. Only functional categories with at least one significant (P < 0.0001; one-tailed binomial test) connection to another category are displayed for each method. Nodes with a black outline indicate functional categories with significant intra-connectivity, nodes lacking an outline indicates functional categories that do not have a significant (P < 0.001) number of edges within a functional category. Lines between functional categories indicate a significant (P < 0.0001) number of edges exist between the genes comprising both function categories. The presence of an ∗ indicates that based on a two-tailed Fisher’s exact test, there is a significantly (P < 0.05) greater proportion of edges when comparing CSE-processed data vs. non-CSE data. (A) Non-CSE WGCNA All, (B) non-CSE WGCNA Consensus, (C) CSE WGCNA All, and (D) CSE WGCNA Consensus. [file Image_3.pdf]

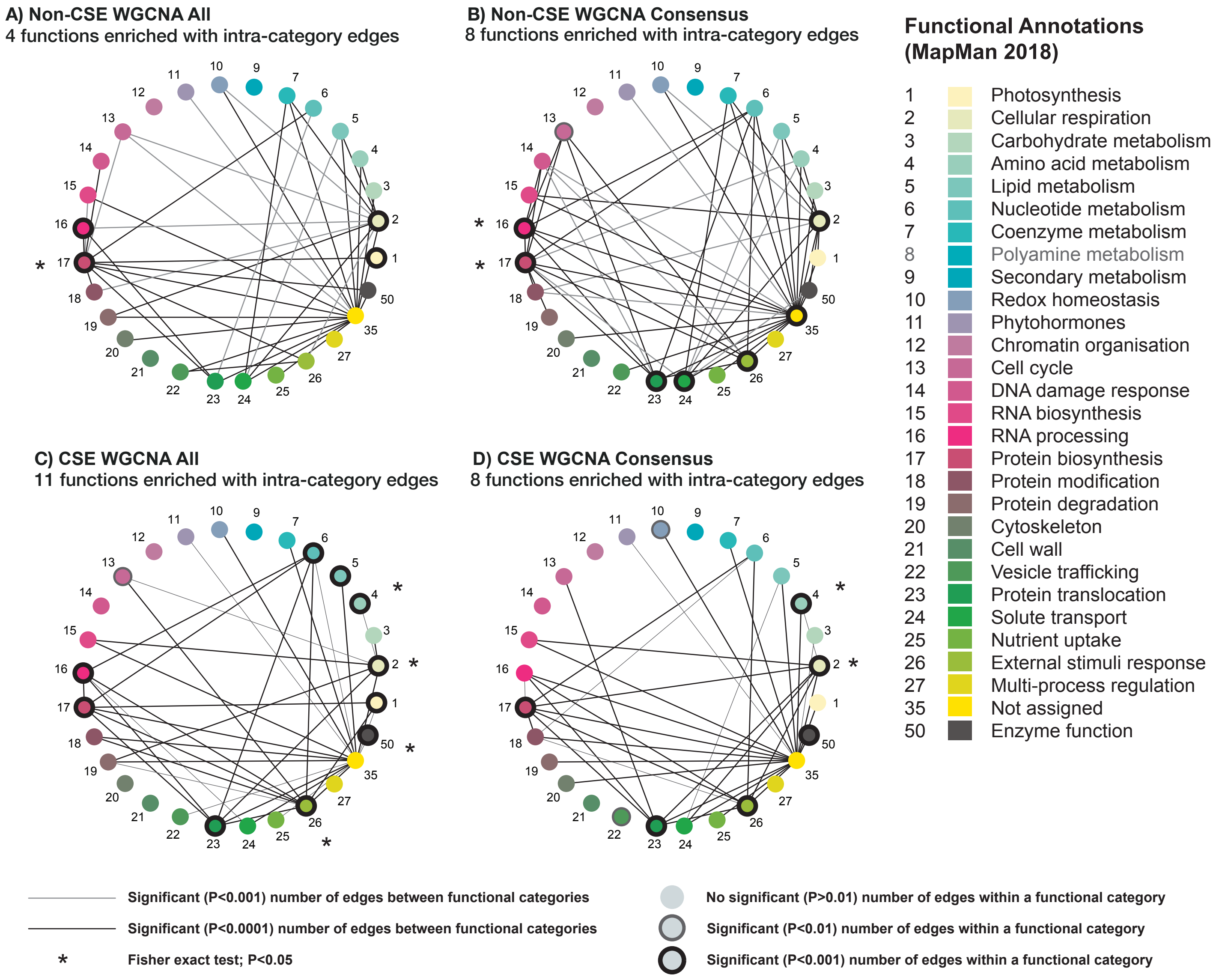

Supplemental Figure 3. Comparative analysis of four different correlation methods based on connectivity between different functional categories in mitochondria. Using newly updated MapMan annotations (MapMan X4 Release 1.0, 2018; Usadel et al., 2009), the mitochondrial set was subdivided into 29 different functional categories. Only functional categories with at least one significant (P<0.0001; one-tailed binomial test) connection to another category are displayed for each method. Nodes with a black outline indicate functional categories with significant intra-connectivity, nodes lacking an outline indicates functional categories that do not have a significant (P<0.001) number of edges within a functional category. Lines between functional categories indicate a significant (P<0.0001) number of edges exist between the genes comprising both function categories. The presence of an * indicates that based on a two-tailed Fisher's exact test, there is a significantly (P<0.05) greater proportion of edges when comparing CSE-processed data vs. Non-CSE data. **(A)** Non-CSE WGCNA All, **(B)** Non-CSE WGCNA Consensus, **(C)** CSE WGCNA All, and **(D)** CSE WGCNA Consensus.
