# Supplementary material for: Centralization Within Sub-Experiments Enhances the Biological Relevance of Gene Co-expression Networks: A Plant Mitochondrial Case Study
Source: Front Plant Sci. 2020 Jun 4;11:524. doi: 10.3389/fpls.2020.00524 (PMC7287149; doi:10.3389/fpls.2020.00524)
Supplement: FIGURE S4 — Synthesis of a conventional co-expression network of Arabidopsis shoots common to four stresses with a CSE Reference Community Set. A core set of stress-responsive genes isolated from non-CSE AtGenExpress stress dataset (Kilian et al., 2007) covering heat, drought, cold, and salt stresses, cross-referenced with the CSE Reference Community. [file Image_4.pdf]

Reference Community	Locus ID	Symbol	ARAPORT11 Description	Functional Enrichment
1	AT2G28000	CPN6-A	chaperonin-60alpha	Photorespiration
1	AT3G13470	CPN6-BETA2	TCP-1/cpn60 chaperonin family protein	
1	AT5G20720	CPN2	chaperonin 20	
1	AT4G33010	GLDP1	glycine decarboxylase P-protein 1	
1	AT2G26080	GLDP	glycine decarboxylase P-protein 2	
1	AT1G11860	-	Glycine cleavage T-protein family	
1	AT5G46800	BOU	Mitochondrial substrate carrier family protein	
1	AT5G35630	GS2	glutamine synthetase 2	
1	AT3G19480	3-PGDH	D-3-phosphoglycerate dehydrogenase	
1	AT5G19760	-	Mitochondrial substrate carrier family protein	
1	AT3G21390	-	Mitochondrial substrate carrier family protein	Thiamin biosynthesis
1	AT5G54770	THI1	thiazole biosynthetic enzyme	
1	AT5G16715	EMB2247	protein EMBRYO DEFECTIVE 2247	
1	AT1G62750	SCO1	Translation elongation factor EFG/EF2 protein	
1	AT4G24280	cpHsc7--1	chloroplast heat shock protein 70-1	
1	AT5G23060	CaS	calcium sensing receptor	
1	AT1G48420	D-CDES	D-cysteine desulfhydrase	
2	AT5G57815	-	Cytochrome c oxidase, subunit Vib family protein	
2	AT2G16710	-	Iron-sulfur cluster biosynthesis family protein	
3	AT5G20180	-	Ribosomal protein L36	Protein synthesis, import and assembly
3	AT3G56070	ROC2	rotamase cyclophilin 2	
3	AT5G40770	PHB3	prohibitin 3	
3	AT1G49410	TOM6	translocase of the outer mitochondrial membrane 6	
3	AT1G18320	-	Mitochondrial import Tim17/Tim22/Tim23 family protein	
3	AT3G46560	TIM9	Tim10/DDP family zinc finger protein	
3	AT3G13860	HSP60-3A	heat shock protein 60-3A	
3	AT3G23990	HSP6	heat shock protein 60	
3	AT3G07770	Hsp89.1	HEAT SHOCK PROTEIN 89.1	
3	AT2G04030	CR88	Chaperone protein htpG family protein	
3	AT1G80270	PPR596	PENTATRICOPEPTIDE REPEAT 596	
3	AT4G26780	MGE2	Co-chaperone GrpE family protein	
3	AT2G35790	-	transmembrane protein	

Reference Community	Locus ID	Symbol	ARAPORT11 Description
4	AT2G05710	ACO3	aconitase 3
4	AT4G10040	CYTC-2	cytochrome c-2
4	AT3G22370	AOX1A	alternative oxidase 1A
4	AT2G34630	GPS1	geranyl diphosphate synthase 1
5	AT5G53350	CLPX	CLP protease regulatory subunit X
7	AT4G36400	D2HGDH	FAD-linked oxidases family protein
9	AT1G07180	NDA1	alternative NAD(P)H dehydrogenase 1
9	AT1G10760	SEX1	Pyruvate phosphate dikinase
9	AT4G11600	GPX6	glutathione peroxidase 6
9	AT1G51610	-	Cation efflux family protein
9	AT5G39410	-	Saccharopine dehydrogenase
9	AT5G10860	CBSX3	Cystathionine beta-synthase family protein
9	AT3G59820	LETM1	LETM1-like protein
9	AT2G41250	-	Haloacid dehalogenase-like hydrolase superfamily protein
21	AT2G14120	DRP3B	dynamin related protein
21	AT3G16230	-	Putative eukaryotic LigT
NP	AT5G58970	UCP2	uncoupling protein 2
NP	AT3G51790	G1	transmembrane protein G1P-related 1
NP	AT5G65720	NFS1	nitrogen fixation S (NIFS)-like 1
NP	AT1G19140	COQ9	ubiquinone biosynthesis COQ9-like protein
NP	AT1G08130	LIG1	DNA ligase 1
NP	AT5G24840	TRM8A	tRNA (guanine-N-7) methyltransferase
NP	AT3G60190	DL1E	DYNAMIN-like 1E
NP	AT5G05520	-	Outer membrane OMP85 family protein
NP	AT5G61810	APC1	Mitochondrial substrate carrier family protein
NP	AT4G27940	MTM1	manganese tracking factor for mitochondrial SOD2
NP	AT1G65420	NPQ7	antigen receptor-like protein (DUF565)
NP	AT1G77670	-	Pyridoxal phosphate dependent transferases superfamily protein
NP	AT5G58070	TIL	temperature-induced lipocalin
NP	AT1G18900	-	Pentatricopeptide repeat superfamily protein
NP	AT5G44730	-	Haloacid dehalogenase-like hydrolase superfamily protein
NP	AT4G12590	-	ER membrane protein complex subunit-like protein
NP	AT3G61440	CYSC1	cysteine synthase C1

Supplemental Figure 4. Synthesis of a conventional co-expression network of Arabidopsis shoots common to four stresses with a CSE Reference Community. A core set of stress-responsive genes isolated from non-CSE AtGenExpress stress dataset (Kilian et al., 2007) covering Heat, Drought, Cold and Salt stresses, cross-referenced with the CSE Reference Community.
