# Supplementary material for: Centralization Within Sub-Experiments Enhances the Biological Relevance of Gene Co-expression Networks: A Plant Mitochondrial Case Study
Source: Front Plant Sci. 2020 Jun 4;11:524. doi: 10.3389/fpls.2020.00524 (PMC7287149; doi:10.3389/fpls.2020.00524)
Supplement: FIGURE S5 — Correlation between the 985 mitochondrion related genes were estimated using Pearson correlation without centralization (Non-Centralized data) and Pearson correlation with CSE preprocessing (CSE preprocessed data). For each approach 484,620 correlations were estimated and the 0.5% (2423) gene correlations with the highest absolute value were used to predict edges in the corresponding gene co-expression network. (A) Estimated density functions over all estimated correlations for non-centralized data (green) and CSE preprocessed data (red). The black line shows the density for correlations estimated on simulated noise. (B) The estimated correlations for the two approaches plotted against each other. Edges shared by both approaches are marked blue (620 (25.6%) of the edges were shared), unique edges for the CSE preprocessing network are marked red, and unique edges for the Non-centralized network are marked green. [file Image_5.pdf]

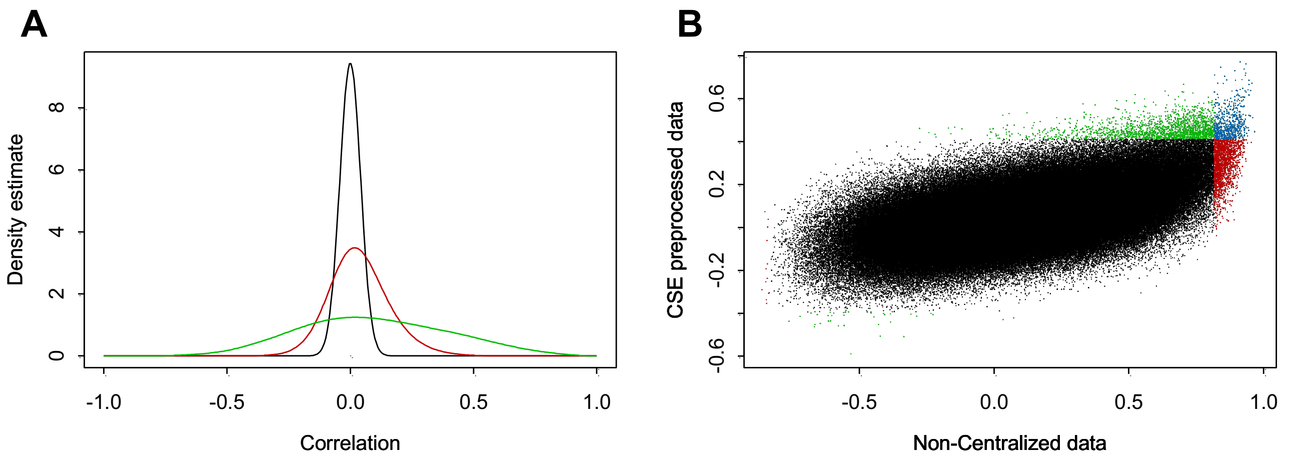

Supplemental Figure 5. Correlation between the 985 mitochondrion related genes were estimated using Pearson correlation without centralization (Non-Centralized data) and Pearson correlation with CSE preprocessing (CSE preprocessed data). For each approach 484,620 correlations were estimated and the 0.5 % (2423) gene correlations with the highest absolute value were used to predict edges in the corresponding gene co-expression network. **(A)** Estimated density functions over all estimated correlations for non-centralized data (green) and CSE preprocessed data (red). The black line shows the density for correlations estimated on simulated noise. **(B)** The estimated correlations for the two approaches plotted against each other. Edges shared by both approaches are marked blue (620 (25.6 %) of the edges were shared), unique edges for the CSE preprocessing network are marked red, and unique edges for the Non-centralized network are marked green.
