# Supplementary material for: Centralization Within Sub-Experiments Enhances the Biological Relevance of Gene Co-expression Networks: A Plant Mitochondrial Case Study
Source: Front Plant Sci. 2020 Jun 4;11:524. doi: 10.3389/fpls.2020.00524 (PMC7287149; doi:10.3389/fpls.2020.00524)
Supplement: TABLE S6 — Comparison of the number of edges observed within the mETC and within the functional categories (WFC) 1-27 using different approaches, with or without CSE. Six methods were included: Pearson correlation, partial correlation, WGCNA, WGCNA Consensus network, BC3Net, and GeneNet; and these were utilized on either non-centralized data (non-CSE) or centralized data (CSE). All networks were fixed to a similar sparsity, each with approximately 2400 edges (0.5%). The number of edges observed for the different approaches and data were compared to the number of edges expected by chance within the mETC and WTC. Here, blue numerals indicate that the observed number was significantly larger (p < 0.05) than expected by chance. For each approach, the performance applying centralized versus non-centralized data were tested. Here, an ∗ indicates that significantly more edges (p < 0.05) were observed when using CSE adjusted data. Finally, the results obtained using Pearson correlation and centralized data (PeCSE) were compared to all other methods. Approaches with significantly fewer (p < 0.05) number of edges than PeCSE were marked with a minus sign ‘−’. [file Table_6.docx]

| **Method** | **Data** | **Number of edges** | |
| --- | --- | --- | --- |
|  |  | **mETC** | **WFC** |
| Expected value (by chance) | - | 33.9 | 84.5 |
| Pearson correlation | Non-CSE | 75 (-) | 428 (-) |
|  | CSE | 223* | 485* |
| Partial correlation | Non-CSE | 114 (-) | 438 |
|  | CSE | 218* | 489* |
| WGCNA | Non-CSE | 0 (-) | 329 (-) |
|  | CSE | 182* (-) | 375* (-) |
| WGCNA | Non-CSE | 0 (-) | 252 (-) |
| consensus | CSE | 160* (-) | 218 (-) |
| BC3Net | Non-CSE | 152 (-) | 295 (-) |
|  | CSE | 151 (-) | 323 (-) |
| GeneNet | Non-CSE | 31 (-) | 106 (-) |
|  | CSE | 23 (-) | 105 (-) |

**Supplemental Table 6: Comparison of the number of edges observed within the mETC and within the functional categories (WFC) 1-27 using different approaches, with or without CSE.** Six methods were included: Pearson correlation, partial correlation, WGCNA, WGCNA consensus network, BC3Net and GeneNet; and these were utilized on either non-centralized data (Non-CSE) or centralized data (CSE). All networks were fixed to a similar sparsity, each with approximately 2400 edges (0.5%). The number of edges observed for the different approaches and data were compared to the number of edges expected by chance within the mETC and WTC. Here, blue numerals indicate that the observed number was significantly larger (p<0.05) than expected by chance. For each approach, the performance applying centralized versus non-centralized data were tested: an ‘ * ‘ indicates that significantly more edges (p<0.05) were observed when using CSE adjusted data. Finally, the results obtained using Pearson correlation and centralized data were compared to all other methods. Approaches with significantly fewer (p<0.05) number of edges than Pearson correlation with centralized data were marked with a minus sign ‘ - ‘.
